# Comprehensive Evaluation of Fundamental Motor Skills: Insights From the Test of Gross Motor Development-3

**DOI:** 10.7759/cureus.46381

**Published:** 2023-10-02

**Authors:** Suresh J, Subash S

**Affiliations:** 1 Physiotherapy, Sri Ramaswamy Memorial (SRM) College of Physiotherapy, Sri Ramaswamy Memorial (SRM) Institute of Science and Technology, Chennai, IND; 2 Pediatrics, Sri Ramaswamy Memorial (SRM) Medical College Hospital and Research Centre, Sri Ramaswamy Memorial (SRM) Institute of Science and Technology, Chennai, IND

**Keywords:** fundamental motor skills, motor skills, children’s development, locomotor subset, test of gross motor development (tgmd)

## Abstract

Background: A comprehensive evaluation of basic motor abilities is provided by the Test of Gross Motor Development-3 (TGMD-3), which assesses 13 basic motor skills. These skills are categorized into locomotor and ball skill subsets.

Objective: An assessment of gross motor skills in diverse populations with the TGMD-3 is the goal of this study.

Methods: From control and intervention groups, locomotor subset scores were collected. In addition to identifying developmental delays, the study highlights the TGMD-3's ability to detect deficits in motor skills. Depending on whether a skill meets the criteria, it is scored as either a 1 or a 0. A locomotor score of 46, a ball skill score of 54, and an overall gross motor score of 100 are the maximum scores.

Results: The control group's baseline scores increased from 34.3±0.9 to 37.3±0.7 by the sixth week (p = 0.03), while the intervention group's scores rose from 36.5±1.1 to 40.9±0.6 (p = 0.0007). Both groups also showed similar trends in gross motor coordination scores.

Conclusion: Gross motor skill assessment is robust across different populations, making the TGMD-3 an effective tool for improving motor development and performance.

## Introduction

In order for a child to grow and thrive, he or she must develop physical skills and acquire motor skills. Exploring the environment, participating in physical activities, and developing healthy habits for life are all dependent on these skills [1]. Educators, healthcare professionals, researchers, and parents all have a stake in assessing and understanding gross motor skills. A gross motor skill involves movements involving large muscle groups of the body, such as moving through space, maintaining stability, and manipulating objects [2]. Not only do these skills contribute to physical proficiency, but they also improve cognitive, social, and emotional abilities [3].

In addition to providing valuable insights into children's gross motor skills and developmental progress, the Test of Gross Motor Development-3 (TGMD-3) has been well-established and widely recognized. With TGMD-3, you can evaluate these skills across different age groups using a comprehensive framework designed by specialists in the field [2]. A further advancement in developmental research, pedagogical approaches, and assessment methods is incorporated into this assessment in comparison to its predecessors, the TGMD and TGMD-2.

As a locomotor and object control assessment tool, the TGMD-3 has a number of key strengths. Run, jump, and throw are locomotor skills, while object control skills include actions like catching and throwing [1]. It is crucial for a child to be proficient in these skills not only for their physical development but for their active participation in sports, recreational activities, and everyday life as well [4].

Research studies can be conducted using the TGMD-3 to measure gross motor skills in a standardized and reliable manner. Children's development can be studied in various ways by researchers, such as motor skill acquisition, the effectiveness of physical activity interventions, developmental milestones, and the influences of factors such as gender, age, and cultural background [5].

As an evidence-based practice tool, the TGMD-3 is valuable due to its validity and reliability. Research and education programs targeting specific aspects of gross motor development can be tailored based on assessment results. Furthermore, the information gained from TGMD-3 assessments contributes to a better understanding of children's health and well-being through physical activity, health, and wellness promotion.

## Materials and methods

The Institutional Review Board, denoted as MCH and RC of SRM, in conjunction with the Institutional Ethics Committee, known as IST of SRM, granted their formal sanction for this study. Moreover, the study's documentation and protocols were duly registered and recorded in the Clinical Trials Registry-India. The assigned CTRI registrant number for this study is CTRI/2021/09/036196. The Ethics Clearance Number provided for this research initiative is 1911/IEC/2020.

Protocol of the study

Informed consent was diligently acquired from either the parent or the legal guardian, in instances where the parent was unavailable or inaccessible. The selection of subjects was carried out meticulously, adhering to predefined inclusion and exclusion criteria. Subsequently, the chosen subjects underwent a rigorous screening process. To ensure unbiased and methodical allocation, a computerized randomization procedure was employed, effectively categorizing the participants into distinct control and test groups.

Criteria for selection of subjects

Subjects represented in the study were boys and girls. A borderline impaired or delayed patient was defined as one with a Gross Motor Development-Third Edition score spanning 70 to 79. With this comprehensive approach, the effectiveness of the assessment tool could be assessed across a range of individuals with varying gross motor development levels.

Subjects who were affected with neurological or movement disorders, congenital or musculoskeletal conditions that affect motor function, visual or sensory impairments, cognitive deficits, epilepsy history, ongoing physical therapy, and hearing impairment were excluded from the study. The study's motor performance assessments were exempted from such confounding factors to ensure data integrity.

Study group

G-1 

Forty-four children were selected based on the inclusion and exclusion criteria. Conventional gross motor training was used as the control group. In addition to running, catching, jumping, and ball-related skills like throwing, catching, and kicking, this training regimen aimed to increase core strength and mobility. It was encouraging to see that the children who took part in the training pattern achieved positive results.

G-2

Forty-five children were selected based on the inclusion and exclusion criteria. A virtual reality gaming program was facilitated for six weeks by G-2, the interventional group. A 45-minute session was conducted each day for participants within this group, three times per day.

A comprehensive gross motor training program included strength exercises, balance drills, and coordination drills. A TGMD-3 assessment was conducted at the baseline visit as part of the initial evaluation of motor coordination. Progress and outcomes were tracked through TGMD-3 tests conducted at 2, 4, and 6 weeks following the initial assessment.

The TGMD-3 test consists of 13 fundamental motor skills that are assessed by the TGMD-3 tool, which was developed to assess basic motor skills. There are two subsets of this scale: locomotor skills and ball skills. In addition to detecting delays in gross motor development, this tool is capable of identifying deficits in gross motor development. Each skill performed by the subject was scored using the tool. In the case that the criteria were met, a score of 1 was given. In the absence of such criteria, a score of 0 was given. The maximum score for the locomotor subset is 46, the maximum score for the ball skill subset is 54, and the maximum score for overall gross motor performance is 100 [6].

Statistical analysis

All presented values are expressed as mean ± standard deviation (SD). The ANOVA test was employed to analyze all conducted assessments. Intragroup and intergroup result comparisons were computed and interpreted, with statistical significance attributed to p-values below 0.05.

## Results

TGMD-3 test

To assess the coordination of the subjects, the TGMD-3 tool was used, which contains two subsets for analysis, namely, locomotor and ball skills. A mean ± SD is calculated for each result.

Locomotor subset

**Figure 1:**
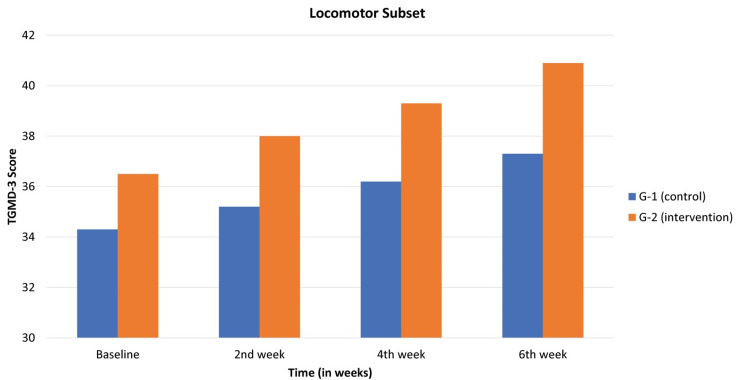
At baseline and second, fourth, and sixth weeks, TGMD-3 assessment showing locomotor scores between control and intervention groups TGMD-3: Test of Gross Motor Development-3

The locomotor subset scores were documented for both the control and intervention groups. In the control group, baseline scores were 34.3±0.9, escalating to 35.2±0.9 in the second week, 36.2±0.8 in the fourth week, and reaching 37.3±0.7 by the sixth week. This progression exhibited statistical significance, with a p-value of 0.03. Meanwhile, the intervention group displayed baseline scores of 36.5±1.1, surging to 38±1.05 in the second week, 39.3±0.9 in the fourth week, and culminating at 40.9±0.6 by the sixth week. This advancement also demonstrated statistical significance, supported by a p-value of 0.0007. The result of the locomotor subset was observed in Table 1 and Figure 1.

**Table 1:** Locomotor subset expressed as mean ± SD

Subset	Study Groups	Baseline	2nd week	4th week	6th week	p-value
Locomotor subset	G-1 (control)	34.3±0.9	35.2±0.9	36.2±0.8	37.3±0.7	0.03
	G-2 (intervention)	36.5±1.1	38±1.05	39.3±0.9	40.9±0.6	0.0007

Ball skill subset

**Figure 2:**
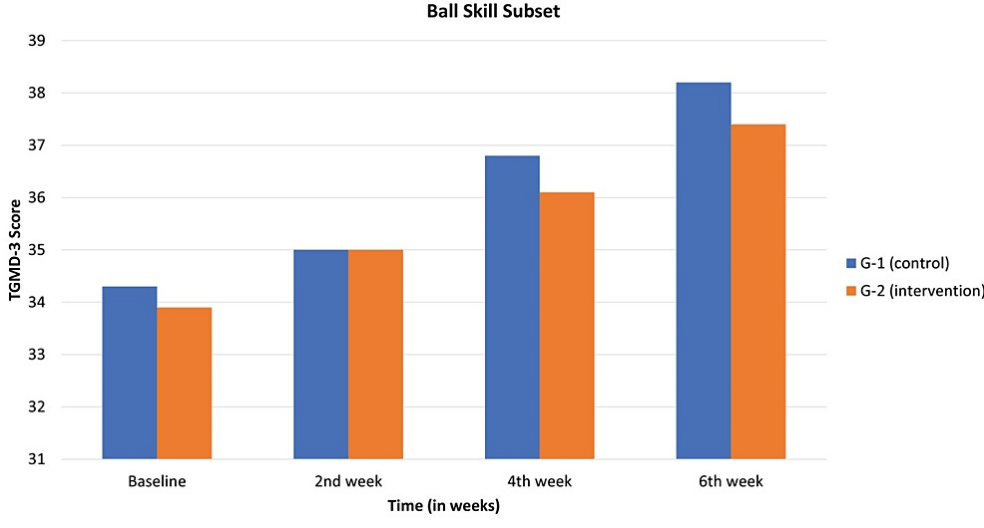
In the TGMD-3 assessment, control and intervention groups are compared at baseline and second, fourth, and sixth weeks for the ball skill subset

In Table 2 and Figure 2, the TGMD-3 assessment for the ball skill subset was observed. The control group exhibited baseline results of 34.3±1.4, which progressed to 35±1.3 in the second week, 36.8±1.2 in the fourth week, and finally reached 38.2±1.2 by the sixth week. This observed trajectory displayed statistical significance, denoted by a p-value of 0.04. Conversely, the intervention group showcased baseline results of 33.9±0.4, which slightly increased to 35±0.6 in the second week, further advancing to 36.1±0.6 in the fourth week and culminating at 37.4±0.5 by the sixth week. These results also exhibited statistical significance, substantiated by a p-value of 0.009.

**Table 2:** TGMD-3 expressed as mean ± standard deviation (SD) for the ball skill subset TGMD-3: Test of Gross Motor Development-3

Subset	Study Groups	Baseline	2nd Week	4th Week	6th Week	p-value
Ball skill subset	G-1 (control)	34.3±1.4	35±1.3	36.8±1.2	38.2±1.2	0.04
	G-2 (intervention)	33.9±0.4	35±0.6	36.1±0.6	37.4±0.5	0.009

Overall gross motor coordination

**Figure 3:**
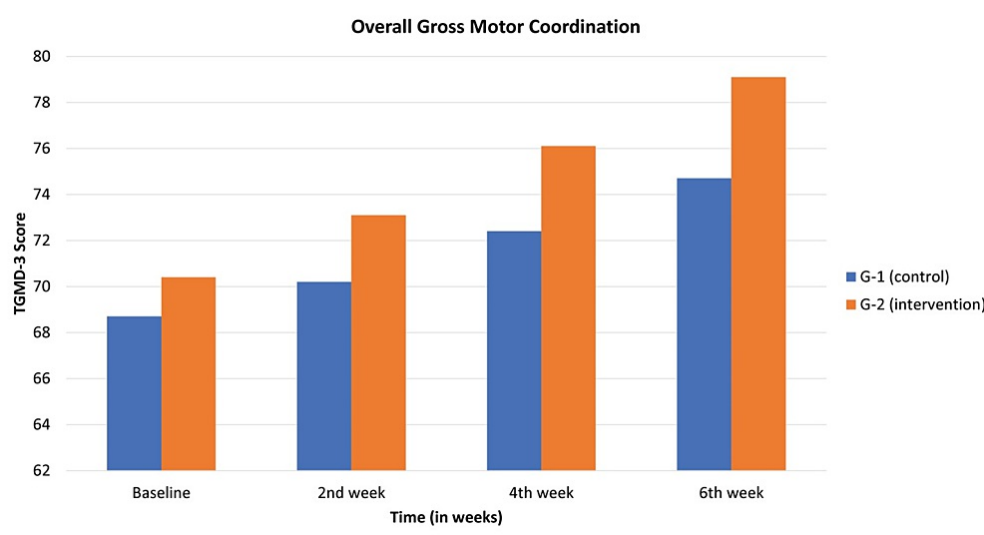
Means and standard deviations for gross motor coordination score TGMD-3 TGMD-3: Test of Gross Motor Development-3

The overall gross motor coordination score was analyzed, and the observed values were recorded in Table 3 and Figure 3. The composite gross motor coordination score, a sum of the locomotor subset and ball skill subset, was evaluated. In the control group, baseline scores were 68.7±1.7, escalating to 70.2±4.8 in the second week, 72.4±0.9 in the fourth week, and reaching 74.7±0.8 by the sixth week. A statistically significant association was observed, with a p-value of 0.007. In the interventional group, baseline scores were 70.4±1.2, surging to 73.1±1.2 in the second week, 76.1±1.5 in the fourth week, and culminating at 79.1±1.2 by the sixth week. Notably, a p-value of 0.0009 indicated statistical significance.

**Table 3:** Means and standard deviations for gross motor coordination score TGMD-3 TGMD-3: Test of Gross Motor Development-3

Subset	Study groups	Baseline	2^nd^ week	4^th^ week	6^th^ week	p value
Overall gross motor coordination	G-1 (control)	68.7±1.7	70.2±4.8	72.4±0.9	74.7±0.8	0.007
	G-2 (intervention	70.4±1.2	73.1±1.2	76.1±1.5	79.1±1.2	0.0009

## Discussion

In a comparative study, the effectiveness of both the traditional TGMD-3 protocol and a modified version with visual support was examined [6]. Results indicated that both protocols effectively measure gross motor performance in typically developing children and those with developmental coordination disorder.

This underscores the utility of both forms of TGMD-3 for assessing gross motor skills across diverse populations. Recent studies have examined the TGMD-3 skills item difficulty [7,8], but they have all focused on the items and not the criteria that comprise the items.

As a result of the ball skill subscale having eight additional performance criteria compared with the locomotor subscale, the ball skill subscale had an overall higher average raw score than the locomotor subscale [9]. Researchers evaluated internal consistency to ensure that TGMD-3 variables are homogeneous [10]. This assessment was highly reliable, as evidenced by its high test-retest reliability and internal consistency. There was ample evidence that the version of this assessment used by test users with confidence has a reliable, appropriate design.

Among the new skills in the TGMD-3, three were added and two were removed compared to previous editions of the TGMD-2 and TGMD-3. Compared to the previous assessment version, the factor loadings for the TGMD-3 appear to be high due to these skill changes. As a result of its two-factor structure, the TGMD-2 separates locomotor skills from object control skills [11]. Based on reliability and validity measures, the TGMD-3 could be used and interpreted as intended.

To ensure that the TGMD-3 mimics rolling a ball as closely as possible, underhand toss was strategically selected as the closest skill to rolling a ball (since going from TGMD-2 to TGMD-3 required removing the rolling skill).

It was reported by a study that the TGMD-2 and the MABC (first edition) should be used to assess gross motor skills in children with developmental coordination disorder [12]. However, further evidence of validity is needed for the MABC. A detailed review of gross motor assessment tools for children was published [13]. These tools were not assessed for validity, reliability, or responsiveness to changes. There are several publications reporting factor analyses of the TGMD-2, which is the only assessment tool with established construct validity. The consistency of TGMD-2's psychometric properties, as established in prior studies, suggests that the updated TGMD-3 likely maintains its reliability, making it a dependable tool for assessing gross motor skills [14,15].

Notably, the prevalence of baseball in the United States may contribute to greater proficiency in specific TGMD-3 skills, such as the two-hand strike, compared to regions where other sports, like tennis, dominate. Cultural and contextual factors can influence motor skills performance, as evidenced by lower internal consistency in the TGMD-3 ball skill subtest among typically developing participants, a phenomenon similarly reported [15]. Further research utilizing the Fugl-Meyer Assessment for Upper Extremity has explored the impact of interventions on upper limb motor function. Several studies reveal significant differences among intervention groups, although muscle strength improvements remain inconclusive [14,15]. A study investigation in 2021 examined the effects of wrist weights during strength, velocity, and resistance (SVR) training on upper limb muscle strength, revealing significant improvements [15]. Similar findings have been reported previously. Incorporating wrist weights into SVR training showcases its positive impact, making it a valuable technique for enhancing upper limb performance and strength across various populations, including athletes, rehabilitation patients, and individuals seeking to improve their upper limb capabilities.

Limitations

The evidence highlighted in this study must also be observed from the perspective of its limitations. The first limitation is related to the sample enrolled. It was composed of neurological developing children. Another limitation is the lack of previous studies examining item psychometrics, which restrains our capacity for comparison with other samples. Furthermore, we are unable to compare our results with other samples because no previous studies have evaluated item psychometrics. This is the first investigation of the brief version of the TGMD-3 for individuals with VI to our knowledge. This inquiry has other strengths in addition to its innovation. To be more specific, we accounted for item difficulty as well as discrimination in our analyses. Furthermore, we examined convergent validity and composite reliability of latent traits related to locomotor and object control skills. The robust analyses (namely composite reliability and variance extracted) take into account the inherent risk of running multiple models on a single sample, thereby mitigating some of it.

## Conclusions

As a comprehensive tool for evaluating fundamental motor skills, the TGMD-3 categorizes 13 skills into locomotor and ball subsets. In addition to detecting developmental delays, the TGMD-3 accurately detected deficits in motor skills through a 0 to 1-point scoring system. Locomotor subsets rated 46 points; ball skill subsets rated 54 points; and overall gross motor performance rated 100 points. In both control and intervention groups, locomotor subset scores showed significant improvements, as did composite gross motor coordination scores. Collectively, these results demonstrate the reliability of the TGMD-3 as a tool for assessing gross motor skills across diverse populations.

## References

[1] Haywood KM, Getchell N (2021). Life Span Motor Development. https://books.google.co.in/books?hl=en&lr=&id=onkvEAAAQBAJ&oi=fnd&pg=PP11&dq=Life+span+motor+development&ots=CBSV3HHxu5&sig=sgulSw7pa1SP_9ktA6aQIukXFxs&redir_esc=y#v=onepage&q=Life%20span%20motor%20development&f=false.

[2] Ulrich DA (2000). Test of Gross Motor Development 2. https://www.researchgate.net/publication/283530031_Test_of_gross_motor_development-2.

[3] Lubans DR, Morgan PJ, Cliff DP, Barnett LM, Okely AD (2010). Fundamental movement skills in children and adolescents: review of associated health benefits. Sports Med.

[4] Clark JE, Humphrey J (2002). The mountain of motor development: A metaphor. Motor Dev Res Rev.

[5] Robinson LE, Goodway JD (2009). Instructional climates in preschool children who are at-risk. Part I: Object-Control Skill Development. Res Q Exerc Sport.

[6] Allen KA, Bredero B, Van Damme T, Ulrich DA, Simons J (2017). Test of Gross Motor Development-3 (TGMD-3) with the use of visual supports for children with autism spectrum disorder: validity and reliability. J Autism Dev Disord.

[7] Barnett LM, Minto C, Lander N, Hardy LL (2014). Interrater reliability assessment using the Test of Gross Motor Development-2. J Sci Med Sport.

[8] Kim S, Kim MJ, Valentini NC, Clark JE (2014). Validity and reliability of the TGMD-2 for South Korean children. J Mot Behav.

[9] Valentini NC (2012). Validity and reliability of the TGMD-2 for Brazilian children. J Mot Behav.

[10] Simons J, Hombeeck CV (2003). Toepasbaarheid van de Test of Gross motor Development Second Edition. Toepasbaarheid van de test of gross motor development.

[11] Ahmad MA, Singh DK, Mohd Nordin NA, Hooi Nee K, Ibrahim N (2019). Virtual reality games as an adjunct in improving upper limb function and general health among stroke survivors. Int J Environ Res Public Health.

[12] Kim JH (2018). Effects of a virtual reality video game exercise program on upper extremity function and daily living activities in stroke patients. J Phys Ther Sci.

[13] Norouzi-Gheidari N, Hernandez A, Archambault PS, Higgins J, Poissant L, Kairy D (2019). Feasibility, safety and efficacy of a virtual reality exergame system to supplement upper extremity rehabilitation post-stroke: a pilot randomized clinical trial and proof of principle. Int J Environ Res Public Health.

[14] Miclaus R, Roman N, Caloian S (2020). Non-immersive virtual reality for post-stroke upper extremity rehabilitation: a small cohort randomized trial. Brain Sci.

[15] Corregidor-Sánchez AI, Segura-Fragoso A, Criado-Álvarez JJ, Rodríguez-Hernández M, Mohedano-Moriano A, Polonio-López B (2020). Effectiveness of virtual reality systems to improve the activities of daily life in older people. Int J Environ Res Public Health.

